# Microfluidic Sensor Based on Cell-Imprinted Polymer-Coated Microwires for Conductometric Detection of Bacteria in Water

**DOI:** 10.3390/bios13100943

**Published:** 2023-10-20

**Authors:** Shiva Akhtarian, Ali Doostmohammadi, Daphne-Eleni Archonta, Garrett Kraft, Satinder Kaur Brar, Pouya Rezai

**Affiliations:** 1 Department of Mechanical Engineering, York University, Toronto, ON M3J 1P3, Canada; shivaakh@yorku.ca (S.A.); doost@yorku.ca (A.D.);; 2 Sixth Wave Innovations Inc., Halifax, NS B4A 0H3, Canada; 3 Department of Civil Engineering, York University, Toronto, ON M3J 1P3, Canada; satinder.brar@lassonde.yorku.ca

**Keywords:** cell-imprinted polymer (CIP), CIP coating, microfluidic, biosensor, bacteria detection

## Abstract

The rapid, inexpensive, and on-site detection of bacterial contaminants using highly sensitive and specific microfluidic sensors is attracting substantial attention in water quality monitoring applications. Cell-imprinted polymers (CIPs) have emerged as robust, cost-effective, and versatile recognition materials with selective binding sites for capturing whole bacteria. However, electrochemical transduction of the binding event to a measurable signal within a microfluidic device to develop easy-to-use, compact, portable, durable, and affordable sensors remains a challenge. For this paper, we employed CIP-functionalized microwires (CIP-MWs) with an affinity towards *E. coli* and integrated them into a low-cost microfluidic sensor to measure the conductometric transduction of CIP–bacteria binding events. The sensor comprised two CIP-MWs suspended perpendicularly to a PDMS microchannel. The inter-wire electrical resistance of the microchannel was measured before, during, and after exposure of CIP-MWs to bacteria. A decline in the inter-wire resistance of the sensor after 30 min of incubation with bacteria was detected. Resistance change normalization and the subsequent analysis of the sensor’s dose-response curve between 0 to 10^9^ CFU/mL bacteria revealed the limits of detection and quantification of 2.1 × 10^5^ CFU/mL and 7.3 × 10^5^ CFU/mL, respectively. The dynamic range of the sensor was 10^4^ to 10^7^ CFU/mL where the bacteria counts were statistically distinguishable from each other. A linear fit in this range resulted in a sensitivity of 7.35 μS per CFU/mL. Experiments using competing *Sarcina* or *Listeria* cells showed specificity of the sensor towards the imprinted *E. coli* cells. The reported CIP-MW-based conductometric microfluidic sensor can provide a cost-effective, durable, portable, and real-time solution for the detection of pathogens in water.

## 1. Introduction

There has been an increasing demand for the inexpensive, rapid, and portable monitoring of pathogenic and indicator bacteria in medical and environmental samples [1,2]. The conventional methods of detecting bacteria include immunological assays, molecular tests, and cell culturing, which are labor-intensive, time-consuming, and expensive while requiring trained personnel and complex equipment [3]. Point-of-care (PoC) devices have emerged as promising tools to enable the inexpensive, rapid, and on-site sensing of biological contaminants with high selectivity, sensitivity, and reproducibility [4]. PoC sensors rely on various transduction modalities that are resulted from the specific binding of target bacteria to different bio-recognition materials that are discussed below [5]. 

PoC devices use various transduction techniques such as electrochemiluminescence [6,7], surface plasmon resonance (SPR) [8], fluorescence [9], colorimetry [10], mass spectrometry using quartz crystal microbalance (QCM) [11], and electrochemistry [12]. Electrochemical detection has multiple advantages in terms of high sensitivity and specificity, rapid measurement, and being inexpensive and suitable for the miniaturization for on-site testing [2,13]. Numerous electrochemical bacteria sensors with various bio-recognition elements such as antibodies, aptamers, and bacteriophages have been developed to date [14,15]. Despite offering excellent detection performances in lab testing, deployment of these sensors is challenging due to the instability of these bio-recognition materials under varying temperatures and moisture contents in real field environments. 

Molecularly imprinted polymers (MIPs), also called “synthetic antibodies”, are polymeric recognition materials with a robust and competitive affinity to target molecules like their bio-recognition counterparts and the added value of polymers’ stability in harsh environments [16,17]. MIPs are synthesized in the presence of a target analyte like a protein, referred to as the template during fabrication. The utilized template’s physical (morphological) and chemical (functional) properties are retained in the generated cavities after polymerization and template removal from the MIP matrix. This allows MIPs to selectively rebind to the same targets in complex biological fluids and environmental samples [18]. 

As a versatile group of compounds, MIPs can recognize analytes of many sizes, shapes, and chemical functionalities, like proteins, membrane glycolipids, and even whole bacteria cells [19,20,21,22,23], where they are referred to as cell-imprinted polymers (CIPs). The choice of appropriate CIP composition and its functional monomers (FMs) defines the CIPs–template interaction and directly affects the affinity of the formed recognition sites towards target cells [18]. The effective imprinting of biological cells is reported to be achieved by choosing FMs with proper side chains [24], enabling the formation of non-covalent bonds with templates. This is attained through weak interactions, i.e., hydrogen bonds and van der Waals interactions combined with shape complementarity between CIP cavities and the target cells [24,25,26,27,28]. CIPs synthesized by copolymers of methacrylic acid (MAA), acrylamide (AAM), methyl methacrylate (MMA), and N-vinylpyrrolidone (VP) have demonstrated enhanced selectivity toward biological templates by providing additional side chains during non-covalent imprinting [25,26,28,29]. We used this composition in previous works and generated microspheres with CIP shells [30] and CIP-coated microwires (called CIP-MWs) [31] that could bind to *E. coli* with high efficiency. 

Electrochemical sensors have been successfully integrated with MIPs and used with various readout methods including field-effect transistors, conductometry, electrochemical impedance spectroscopy (EIS), voltammetry, and amperometry. These platforms convert the interaction between MIPs and target molecules on an electrode surface into various electrical signals [32]. Conductometric measurement involves monitoring the change in conductivity of the MIP receptor layer over time in response to the MIP binding to its complementary analyte [32], which changes the concentration of ionic species at the interface of liquid–solid [33]. This approach employs direct current instead of alternating current, as in the EIS method, and has the potential to be advantageous in the creation of fast, compact, and portable biosensors that are powered by a battery in the future. Furthermore, the DC approach does not require a reference electrode and does not have the complications associated with selecting an appropriate equivalent circuit model for analyzing the frequency-dependent impedance in the EIS method, which could cause non-accurate results in electrode–electrolyte systems [34]. Additionally, AC measurements usually require longer times and a large volume of solution (>5 mL) for reliable measurements [34]. However, electrolysis could affect the resistance measurements in DC methods by generating bubbles on electrodes or changing the ion concentration of the liquid. Thus, this method can only be applied to liquids with low ion concentrations (low conductivity) [35] such as drinking water [36].

Microfluidics has enabled the fabrication of field-deployable and low-cost sensors, which would need a small sample volume and a short time for biodetection [37]. Various MIP-based microfluidic devices to electrochemically detect target molecules have been reported to date [38,39]. However, using CIPs in microfluidic electrochemical sensors to detect whole cells remains a technological gap. In this paper, we report the integration of CIP-MWs into a low-cost conductometric microfluidic sensor to transform the binding of CIP cavities to target *E. coli* bacteria into a quantitative electrical signal. By monitoring the resistance of bacteria suspensions with various cell counts in a microchannel between two suspended CIP-MWs, the effect of captured cells by the CIP coatings on the conductance signal was studied. The developed sensing platform can be integrated into a hand-held device in the future, enabling the on-field and low-cost monitoring of pathogens.

## 2. Material and Methods

### 2.1. Materials

All chemicals including acetonitrile, methanol, sulfuric acid, acetic acid, tetraethoxysilane, phosphate-buffered saline (PBS), sodium chloride (NaCl), 2, 2′-azoisobutyronitrile (AIBN), ethylene glycol dimethacrylate (EGDMA), methylmethacrylate (MMA), N-vinylpyrrolidone (VP), acrylamide (AAM), methacrylic acid (MAA), Luria Broth (LB), and LB Broth with agar were purchased from Sigma-Aldrich (St. Louis, MO, USA). Stainless steel microwires (SS-MWs, 125.3 ± 0.5 µm dia., Type 304, Product No.: 40944BZ, Thermo Scientific™, Waltham, MA, USA) were used as CIP coating substrates and electrodes in the microfluidic device. Bacteria strains including *E. coli* OP50, *Sarcina lutea* (#155420), and *Listeria innocua* (#33090) were obtained from the Caenorhabditis Genetics Center (University of Minnesota, USA), Carolina Biological Supply Company, (Burlington, NC, USA), and American Type Culture Collection (ATCC, Cheyenne, WY, USA), respectively. 

### 2.2. Bacteria Culturing and Sample Preparation

All the bacterial strains were cultured in LB liquid growth medium overnight inside a shaker incubator at 37 °C and 150 rpm. The supernatant was removed after centrifugation at 7000× *g* for 15 min. The pellet was resuspended into fresh PBS (pH 7.0). Plate culturing and colony counting were used to determine the bacteria count when needed [40,41]. 

For obtaining the bacteria sample for sensor characterization, 3 ppm NaCl in deionized water was used as a non-fatal [42] carrier electrolyte to resemble drinking waters [43] with very low salinity. For this, 3 mg of NaCl was dissolved in 1 L of deionized (DI) water. The bacterial suspension containing 10^9^ CFU/mL in fresh PBS was centrifuged at 7000× *g* for 15 min, and the pellet was resuspended in the 3 ppm NaCl solution. This process was repeated three times to remove excess PBS. Serial dilution of the bacteria suspension in 3 ppm NaCl solution was performed to obtain the lower bacteria counts for dose–response investigations of the sensor. Trypan blue staining of bacteria exposed to 3 ppm NaCl electrolyte for 30 min indicated that the cells were viable after the exposure. 

To examine bacteria capturing by functionalized MWs within the microfluidic device, green fluorescent protein (GFP)-tagged *E. coli* cells and fluorescent microscopy were used. The mean green intensity of the images was measured with RGB measure plus plugin of Image J software (version 1.54e) and used for comparing the fluorescent intensity.

### 2.3. Surface Functionalization of SS-MWs

MIPs have been synthesized on various flexible metals as supporting substrates including gold, silver, platinum, copper, titanium, and stainless steel (SS). Among these, SS has gained great interest due to its low cost, non-toxicity, corrosion resistance, durability, and rigidity [44]. Furthermore, the surface modification of stainless steel is well developed for establishing a stable chemical bonding with the MIP composition of our work [45]. SS-MWs with 4 cm of length were immersed in acetone, ultrasonicated for 5 min, and then washed with methanol and doubly distilled (DI) water to remove the organic chemicals. The MWs were then dried under ambient conditions. To oxidize the surface of SS-MWs, they were immersed in a 2 M sulfuric acid solution for 2 h and then washed with DI water. Silanization was performed in the next step on the hydroxylated surface of SS-MWs by immersing in a solution of tetraethyl orthosilicate (TEOS)-water-methanol (2-1-8 vol%) for 0.5 h. A post-silanization baking step at 150 °C for 2 h was performed to obtain a robust silane layer with high density. Finally, the SS-MWs were rinsed with ethanol three times and dried with an air gun. 

### 2.4. Preparation of CIP-MWs

CIP pre-polymers were prepared by dissolving MAA (180 µL), AAM (21 mg), VP (4.2 µL), MMA (5.2 µL), EGDMA (570 µL), and AIBN (30 mg) in acetonitrile (2.2 mL). These quantities were optimized previously in a design of experiment exercise, leading to form a uniform and stable CIP coating with a thickness of 2.2 ± 0.4 μm on MWs [31]. The solution was ultrasonicated for 2 min in order to remove the dissolved gases. Subsequently, a pre-polymerization step was performed at 65 °C inside an air-circulated gravity convection oven (Heratherm^TM^, Thermo Fisher Scientific, Dreieich, Germany) for 30 min, when the solution color changed from clear to white. An *E. coli* OP50 suspension (375 µL, 10^9^ CFU/mL) was centrifuged at 3000× *g* for 5 min, and the supernatant was removed. The pellet was resuspended into 1.5 mL of pre-polymer solution transferred into a centrifuge tube. The surface-functionalized SS-MWs were then immersed in the tube, and the tube was sealed. Polymerization was performed at 65 °C for 11 h. The first 30 min was performed while tubes were rotating using a tube rotator inside the oven to increase the homogeneity of CIP coating along the surface of the MW. 

After polymerization, the CIP-MWs were washed with methanol-acetic acid (*v*/*v*: 9/1), methanol, and DI water for 20 s to remove bacteria templates from the CIP coatings and generate associated cavities for rebinding. As a control group, non-imprinted polymer (NIP) MWs were prepared simultaneously using an identical synthesis procedure, without adding *E. coli* templates. 

### 2.5. Microfluidic Device

**Figure 1:**
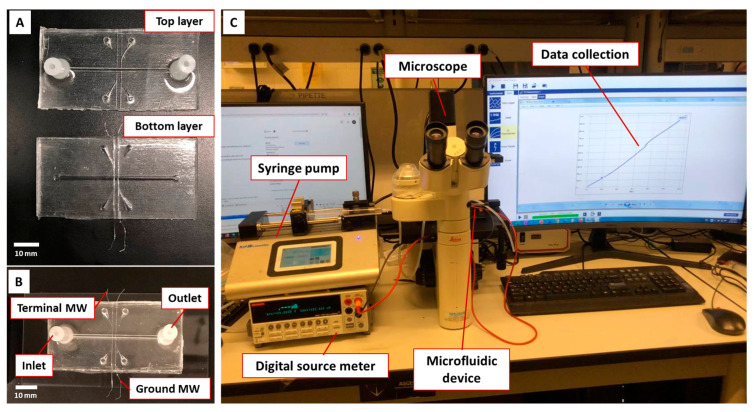
Conductometric microfluidic bacteria sensor and experimental setup. (**A**) Top and bottom PDMS layers with installed MWs for fabrication of microfluidic device, (**B**) final microfluidic device after plasma bonding of two PDMS layers, (**C**) experimental setup showing the instruments required for the sensor characterization.

The microfluidic sensor comprised two mirrored polydimethylsiloxane (PDMS, Sylgard 184 silicone elastomer kit, Dow Corning Co., Midland, MI, USA) layers containing the design of a microchannel (500 μm × 900 μm), the network of inlet–outlet, and two MW channels (130 μm ×130 μm) with an inter-wire distance of 1.5 mm (Figure 1A). Each layer was 5 mm in thickness. The master molds for the replica molding of PDMS were designed using CAD Solidworks software (version 2023) and fabricated using 3D printing (Proto3000, Toronto, ON, Canada). The pre-polymer of PDMS was prepared by mixing PDMS base with its curing agent (weight ratio of 10:1) followed by degassing in a vacuum desiccator for 15 min. The pre-polymer was poured over the master molds, followed by curing on a hot plate at 75 °C for 2 h. After curing, the PDMS layers were peeled off, and CIP-MWs were installed in MW channels on one layer. Using oxygen plasma, the two PDMS layers were bonded together and then bonded to a glass slide (Figure 1B). In control experiments, uncoated MWs, referred to as SS-MWs, as well as NIP-MWs, were also integrated into microfluidic devices. Three replicates of each sensor were fabricated and tested. 

### 2.6. Conductometric Measurement and Experimental Setup

Figure 1C demonstrates the experimental setup for conductometric measurements. A syringe pump (Legato 110, KD Scientific Inc., Holliston, MA, USA) was used to infuse the sample through the microfluidic channel with a flow rate of 0.2 mL/min. A DC electrical source meter (Model 2410, Keithley Instruments Inc., Solon, OH, USA) and its interface software Kickstart (version v2.11.0) installed on a PC were used to measure the electrical resistance across the CIP-MWs. The terminal and ground MWs were connected to the source meter. A current sweep between 10 nA and 1μA with a step size of 10 nA was applied for 110 s during each experiment, and the corresponding voltage across the CIP-MWs was recorded. Using Ohm’s law, the electrical resistance for 100 data points was calculated by dividing the voltage by the applied current values (R = V/I). A DMIL LED conventional inverted fluorescence microscope (Leica, Wetzlar, Germany) was used to monitor the microchannel during experiments. 

The electrical resistance measurement was performed in three stages. In the first stage, referred to as pre-incubation electrolyte 1 wash, 3 ppm NaCl solution as a blank electrolyte was infused through the microchannel, and the measurement was performed. The resistance value obtained at this stage (R_0_) was used as the baseline resistance of the sensor. Next, the bacteria suspension at a known concentration was run through the microchannel for 30 min to allow incubation and conjugation of bacteria with CIP cavities. Subsequently, the second electrical resistance measurement, R_1_, was performed. The last resistance measurement, R_2_, was performed after running a blank electrolyte for the second time (post-incubation electrolyte 2 wash) through the microchannel for 10 min. Measurements were repeated five times at each step and for three replicate devices.

### 2.7. Specificity Experiments

To investigate the specificity of CIP-MWs and the developed sensor, *Listeria* or *Sarcina* bacteria were used as non-target microorganisms, while the template for CIP preparation was *E. coli.* The *E. coli*-imprinted CIP-MWs were installed in the sensor, and the experimental procedure was performed using water samples spiked with *E. coli*, *Listeria*, or *Sarcina* at 10^8^ CFU/mL.

### 2.8. Data Analysis for Sensor Characterization

The obtained R_1_ and R_2_ resistances were normalized by the measured baseline resistance using the fold change method (R_i_/R_0_, i = 1 or 2) to eliminate the variability among devices. The sensor dose–response curve was generated based on the normalized resistance difference method (ΔR/R_0_ = (R_0_ − R_2_)/R_0_). Here, R_1_ was not used as this resistance includes the effect of bacteria attached to the wire but not necessarily captured by the CIP cavities. Experiments at various bacterial counts were performed and the sensor dose–response curve of ΔR/R_0_ versus bacteria count was plotted. 

Minitab 16 statistical software was used for statistical data analysis. A significance level of 0.05 was adopted. The Mann–Whitney U test, also known as the Wilcoxon rank sum test as the nonparametric version of the parametric t-test, was utilized to identify if any two pairs of means were significantly different [46]. 

The dynamic range of the sensor was identified based on the statistical difference of normalized resistance responses between adjacent bacteria counts (*p*-value < 0.05). The linearity of the sensor was determined by line fitting into the dynamic range of the dose–response curve and analyzing the R^2^ values. The limit of detection (LOD) and limit of quantification (LOQ) were determined as bacteria counts with ΔR/R_0_ equal to that of the blank NaCl solution plus 3 and 10 times its standard deviation (SD), respectively. The sensitivity of the sensor in the linear range of the dose–response curve was found based on the slope of the linear range response. 

## 3. Results and Discussion

### 3.1. Bacteria Capturing by Various Microwires inside the Microfluidic Device 

**Figure 2:**
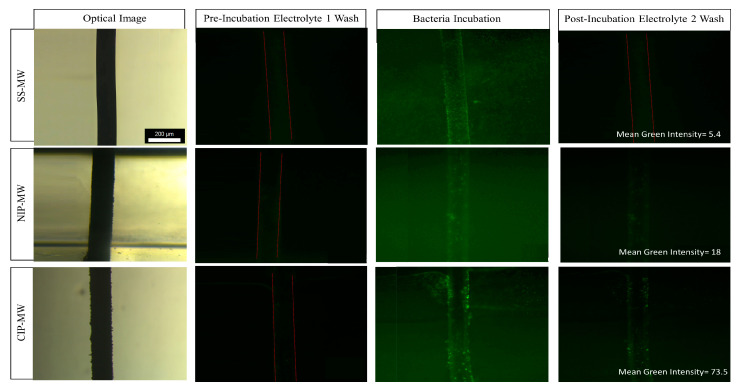
Optical images of the SS-MW, NIP-MW, and CIP-MW (rows) along with their fluorescent images in the microfluidic channel during pre-incubation electrolyte 1 wash, bacteria incubation at 10^8^ CFU/mL for 30 min, and post-incubation electrolyte 2 wash (columns). *E. coli* bacteria were tagged with green fluorescent protein (GFP), hence resulting in a green hue in the channel during the incubation phase and GFP expression spots around the CIP-MW post-incubation. RGB analysis was performed using Image J, and post-incubation green intensities of 5.4, 18, and 73.5 were obtained for SS-MW, NIP-MW, and CIP-MW, respectively.

To examine bacteria capturing by functionalized MWs within the microfluidic device, green fluorescent protein (GFP)-tagged *E. coli* cells and fluorescent microscopy were used. Figure 2 demonstrates the optical and fluorescent microscopy images (10× magnification) of the uncoated SS-MWs, NIP-MWs, and CIP-MWs within the microfluidic device at three stages of the experiment, i.e., pre-incubation electrolyte 1 wash, bacteria incubation at 10^8^ CFU/mL for 30 min, and post-incubation electrolyte 2 wash. Preliminary experiments to study the effect of incubation time on the bacteria capturing of CIP-MWs revealed an optimum incubation time of 30 min, as discussed in Section S1 of the Supplementary Information file. 

When uncoated SS-MWs were installed in the microfluidic device (Figure 2, top row), there was no significant fluorescent signal during the pre-incubation wash. The fluorescent signal increased during the incubation with bacteria, as the GFP-expressing bacteria were infused into the microchannel. However, during the post-incubation wash, the fluorescent signal decreased to near blank (mean green intensity = 5.4) as the GFP bacteria were washed out of the microchannel. 

When a microfluidic device with a pair of NIP-MW electrodes was used (Figure 2, middle row), there was a more intense green light signal after the post-incubation wash (mean green intensity = 18). This revealed that there were still some GFP bacteria present in the region of interest within the device. Comparing with the SS-MWs, these bacteria were observed to be attached to the surface of NIP-MWs due to their non-specific adsorption to the NIP coatings [47].

Conducting this experiment with the microfluidic device that had the CIP-MWs installed as electrodes (Figure 2, bottom row) resulted in a very-high-intensity fluorescent signal after the post-incubation wash with a mean green intensity of 73.5. This higher signal observed for CIP-MWs can be attributed to the enhanced bacteria capturing of the CIP coating related to its imprinted cavities. 

The results of this section visually confirmed a considerable capturing of bacteria on CIP-MWs and enticed us to investigate the detectability of the bacteria using the electrical resistance readout method. 

### 3.2. Conductometric Analysis of the Microfluidic Device

**Figure 3:**
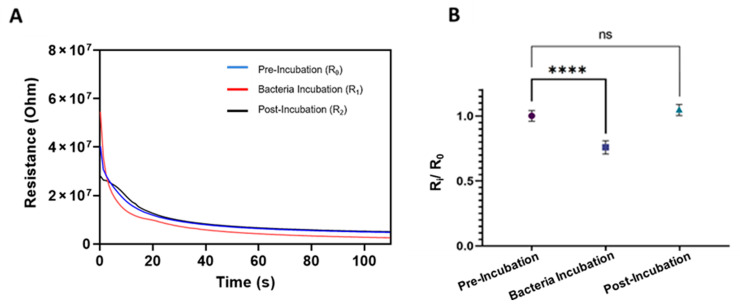
Electrical resistance characterization of SS-MW-based microfluidic sensors. (**A**) Timelapse resistances of one sensor at three stages of pre-incubation wash (R_0_), bacteria incubation at 10^6^ CFU/mL (R_1_), and post-incubation wash (R_2_). (**B**) Averaged normalized resistances (R_i_/R_0_, i = 0, 1, or 2) of three SS-MW-based sensors during three stages of operation. Each measurement was repeated 5 times. Error bars are standard deviations (SD) and ns: non-significant, ****: *p*-value < 0.0001.

After observing the bacteria-capturing characteristics of the SS-MWs, NIP-MWs, and CIP-MWs, we aimed to understand the response time of our sensor in terms of reading a constant electrical resistance upon applying a sweep current in the range of 10 nA–1 μA with a step size of 10 nA. Accordingly, the timelapse resistances of an SS-MW-based microfluidic sensor at three stages of pre-incubation wash (R_0_), bacteria incubation at 10^6^ CFU/mL (R_1_), and post-incubation wash (R_2_) are presented in Figure 3A.

As shown in Figure 3A, the sensor had a transient state at the beginning of the measurement, which was due to the sudden application of an external electric field intrinsic to the measurement system [48]. It also could have been due to a sudden change in the ion concentration around the SS-MWs by applying the current [30]. This transient state lasted for approximately 50 s, after which the resistance between the wires stabilized. 

To obtain the steady-state values of R at each stage of the experiment, the moving average technique [49] was performed to obtain the plateau start point of the resistance curves (see Section S2 of the Supplementary Information File). Based on this analysis, the data points from 55–100, corresponding to 0.55–1 μA current sweep range, were stable and satisfied the plateau criteria in all experiments. Therefore, these data points were used for obtaining the mean and standard deviations (SD) of resistance measurements for further normalization and analysis. 

Repeat experiments for three SS-MW-based sensors were performed, and the average normalized resistances before, during, and after bacteria incubation are plotted in Figure 3B. As shown, the normalized resistance during bacteria incubation was significantly lower (*p*-value < 0.0001) than the baseline resistance. This decrease can be attributed to the presence of suspended bacteria cells between SS-MWs within the microchannel that facilitate the charge transfer between the electrodes. However, the resistance increased to the baseline resistance (R_2_/R_0_~1) after the post-incubation wash, confirming that all the bacteria cells were washed away with the second electrolyte, expectedly.

**Figure 4:**
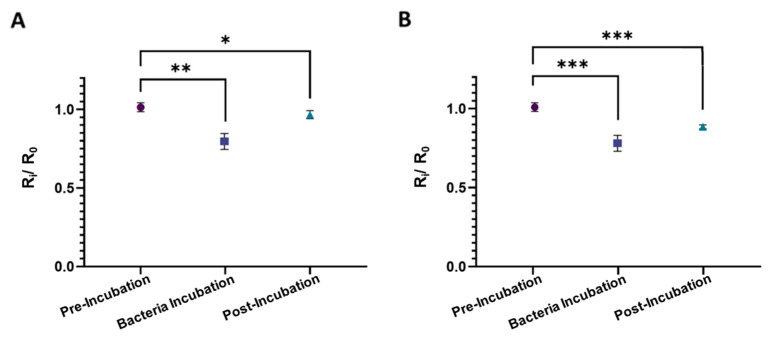
Normalized electrical resistance measurements from the sensors fabricated using (**A**) NIP-MWs and (**B**) CIP-MWs, each in triplicates and with five measurements per condition. Bacteria count during 30 min incubation was 10^6^ CFU/mL. Error bars are standard deviations (SD) and ns: non-significant, *: *p*-value < 0.05, **: *p*-value < 0.01, ***: *p*-value < 0.001.

Figure 4 illustrates the normalized electrical resistance measurements from the sensors fabricated using NIP-MWs and CIP-MWs. As opposed to the results obtained from the uncoated SS-MWs in Figure 3B, when NIP-MWs were used (Figure 4A), the resistance after the post-incubation wash was slightly lower than the baseline resistance (R_2_/R_0_ < 1, *p*-value = 0.012). This decrease in the resistance after washing the bacteria from the microchannel could have been due to the non-specific absorption and attachment of bacteria to the surface of NIP-MWs, which is demonstrated in Figure 2. Interestingly, when CIP-MWs were used in the sensor (Figure 4B), the post-incubation resistance drop became a lot more significant (*p*-value = 0.0008) than the NIP-MWs. The increased normalized resistance drop resulted from the strong attachment of bacteria cells to the CIP-MW cavities that could not be washed away during the post-incubation wash. The results in Figure 3 and Figure 4 clearly show the significant capturing ability of the CIP-MWs and successful transduction of this binding event to measurable electrical signals.

### 3.3. Characterization of the CIP-MW-Based Microfluidic Sensor

**Figure 5:**
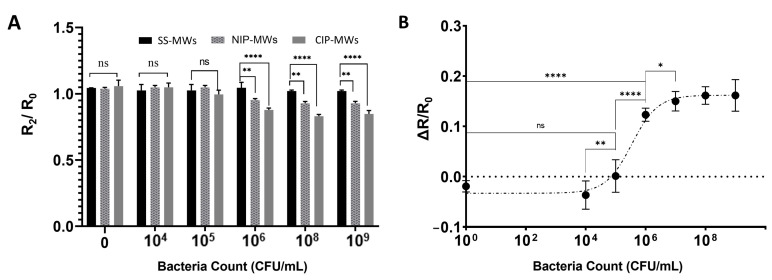
Microfluidic bacteria sensor characterization. (**A**) Normalized post-incubation wash resistance (R_2_/R_0_) response of the microfluidic sensor fabricated with uncoated SS-MWs, NIP-MWs, and CIP-MWs, when exposed to different bacteria counts. (**B**) The dose–response ΔR/R_0_ curve established for the CIP-MWs-based sensor. Error bars are standard deviations (SD) and ns: non-significant, *: *p*-value < 0.05, **: *p*-value < 0.01, and ****: *p*-value < 0.0001.

To further characterize the performance of the CIP-MW-based sensor, the effect of bacteria count on the normalized inter-wire resistance shift of the sensor was studied. *E. coli* bacteria suspensions with cell counts between 0 and 10^9^ CFU/mL were prepared with serial dilution and used in these experiments. Figure 5A shows the normalized post-incubation wash resistance (R_2_/R_0_) of the CIP-MW-based sensor along with the responses obtained from two parallel control experiments, i.e., uncoated SS-MWs and NIP-MWs. It was observed that, for the bacteria counts between 0 and 10^5^ CFU/mL, the CIP sensor did not have a significantly different response than the control experiments (*p*-value > 0.05). However, the difference became more significant by further increasing the bacteria count to 10^6^–10^9^ CFU/mL, demonstrating the dominant effect of CIP coating as compared to NIP coatings or no coating at all. As shown in Figure 5A, the difference between the post-incubation normalized resistance of the CIP sensor with NIP sensor was always less significant (*p*-value < 0.01) than the sensor with uncoated SS-MWs (*p*-value < 0.0001). This can be because of the non-specific adsorption of bacteria to the NIP coatings [50] that can affect the resistance measurement, as shown before.

Figure 5B shows the dose–response ΔR/R_0_ curve established for the CIP-MW-based microfluidics sensor for bacteria counts from 0 to 10^9^ CFU/mL. As can be seen, the dose–response curve of the sensor was non-linear; there were an insignificant response at bacteria concentrations below 10^4^ CFU/mL and a saturation in the normalized resistance shift for bacteria counts higher than 10^7^ CFU/mL. The former effect may have been due to the sensor insensitivity, and the latter effect was due to the high number of bacteria cells surpassing the CIP cavities [50]. The dynamic range of the sensor was determined to be 10^4^–10^7^ CFU/mL due to the significant statistical difference between the subsequent readout signals of the sensor in this range. A linear fit in this range with a goodness of R^2^ = 0.93 resulted in a sensitivity of 7.35 μS per CFU/mL, which is in the range of sensitivities achieved in similar works for the conductometric detection of *E. coli* bacteria using antibodies [51] and aptamers [52]. Based on the 3-sigma and 10-sigma methods, the LOD and LOQ of the CIP sensor were calculated as 2.1 × 10^5^ CFU/mL and 7.3 × 10^5^ CFU/mL, respectively.

The achieved detection range and limit with our established conductometric CIP-MWs-based sensor are comparable with other electrochemical methods for the detection of bacteria using CIPs [53]. However, our proposed sensor has the advantage of the conductometric method’s simplicity and low cost over other electrochemical methods, such as a need for complex instruments as well as a reference electrode [54]. The performance of the developed sensor is also comparable to the performance achieved by coupling other transduction methods with CIPs such as frequency-based sensing techniques using quartz crystal microbalance (QCM) [50,55]. However, conductometric sensing does not have the complexity associated with measuring the frequency change below curtain amounts of bacteria, which was shown to limit the measurable concentration range in related works [50]. The developed sensor can potentially be employed in the detection of bacteria in surface waters that typically contain bacterial cell counts in the range of 10^5^–10^6^ cells/mL [56]. Nevertheless, the detection limit achieved by the developed sensor can be improved by increasing the active surface area of the electrodes by using a microelectrode array to maximize the total surface area and imprinted cavity density available for bacteria binding and detection. Furthermore, a pre-enrichment process can be added to increase the target bacterial concentration for detecting bacteria at lower initial counts, which are going to be investigated in the future.

### 3.4. Specificity of the CIP-MW-Based Microfluidic Sensor

**Figure 6:**
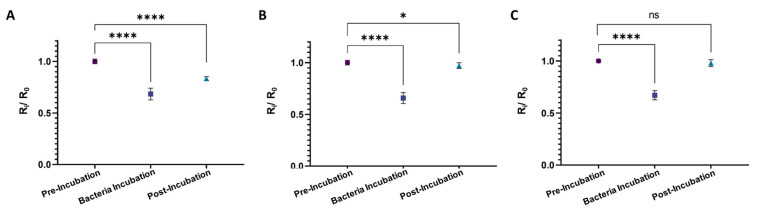
Results of the developed CIP-MWs-based sensor’s specificity test where different microorganisms were used as the target, while the template for CIP preparation was *E. coli*. The target cells are (**A**) *E. coli*, (**B**) *Listeria*, and (**C**) *Sarcina* cells with count of 10^8^ CFU/mL. Measurements were performed in three devices and five measurements per device. Error bars are standard deviations (SD) and ns: non-significant, *: *p*-value < 0.05, and ****: *p*-value < 0.0001.

To investigate the specificity of the CIP-MWs in the developed sensor, *Listeria* and *Sarcina* cells were used as the target microorganism, while the template for CIP preparation was *E. coli.* Figure 6 shows the results performed in triplicates with five repetitions per experiment. As seen in Figure 6A, using *E. coli* as the target microorganism results in a significant (*p*-value < 0.0001) drop in the normalized resistance of the sensor after washing the microchannel with electrolyte 2. However, in the case of using non-template microorganisms as the target, i.e., *Listeria* (Figure 6B) and *Sarcina (*Figure 6C) cells, the difference in the sensor’s pre- and post-bacteria resistance was less significant (*p*-value < 0.05) and insignificant (*p*-value > 0.05), respectively. Considering the size and shape of these two strains, where *Listeria* cells are rod-shaped with approximately 0.5 μm diameter and 0.5–2.0 μm length and *Sarcina* cells are spherical with 1.8–3 μm diameter [57], the small level of significance obtained using *Listeria* cells could be attributed to scarce capturing of these cells by *E. coli* cavities, which are similar in shape and size to the *Listeria* cells rather than the *Sarcina* bacteria. In conclusion, we concluded that our sensor is specific to the bacteria strain, even if cells with similar shapes and morphologies are used in the sensor. In the future, we aim to perform more complex and competitive assays with multiplex water samples.

## 4. Conclusions

A low-cost and miniaturized CIP-based microfluidic sensor for the conductometric detection of bacteria in water was designed and fabricated, and its sensing capacity toward target and non-target bacteria was assessed. The sensor comprised a pair of CIP-MWs installed perpendicularly to a microchannel. The inter-wire DC electrical resistance of the device was shown to significantly decrease after running bacteria suspensions for 30 min through the microchannel in comparison to the corresponding NIP-based sensor. Employing the DC approach eliminated complications associated with AC electrochemical methods such as the need for a reference electrode or selecting an appropriate equivalent circuit model for analyzing the frequency-dependent impedance in the electrochemical impedance spectroscopy (EIS) method, which could cause non-accurate results in electrode–electrolyte systems. Using bacteria suspensions with counts in the range of 0 to 10^9^ CFU/mL, the dose–response curve of the sensor was established. The LOD and LOQ of 2.1 × 10^5^ CFU/mL and 7.3 × 10^5^ CFU/mL were obtained within the dynamic range of 10^4^ to 10^7^ CFU/mL, respectively. A linear fit in this range resulted in a sensitivity of 7.35 μS per CFU/mL. Furthermore, the sensor showed specificity towards the imprinted cells when exposed to other bacteria strains, i.e., *Listeria* and *Sarcina*. The reported sensor detected whole bacteria cells and did not have the complications associated with sample preparation and nucleic acid isolation as required for nucleic acid-based pathogen detection methods. Another advantage was using low-cost and stable CIPs as artificial receptors compared to biological receptors such as enzymes, antibodies, aptamers, and bacteriophages, which are expensive and non-stable in different environmental conditions. In future works, we aim to investigate the effect of non-biological impurities in the detection sample. Also, the reusability of the sensor by running the template removal solution through the device to empty the imprinted cavities will be investigated in the future. The sensor described herein can provide a solution for simple, inexpensive, stable, and selective sensing of bacteria in water at the point of need (PON).

## Data Availability

The data presented in this study are available on request from the corresponding author.

## Supplementary Materials

The following supporting information can be downloaded at: https://www.mdpi.com/article/10.3390/bios13100943/s1, Figure S1: (A) Fluorescent images of CIP-MWs in the microfluidic channel after running buffer (first column) and bacteria suspension in 10 mins intervals. (B) Fluorescent images of CIP-MWs by running only buffer solution at the same time intervals (control experiment); Figure S2: (A) The current vs voltage response obtained on the Kickstart software upon application of current. (B) The corresponding resistance response of the device vs time. Highlighted in purple is the plateau region used to determine the resistance of the device; Figure S3: MATLAB code used to determine the initial plateau.

## References

[1] Rajapaksha P., Elbourne A., Gangadoo S., Brown R., Cozzolino D., Chapman J. (2019). A review of methods for the detection of pathogenic microorganisms. Analyst.

[2] da Silva A.D., Paschoalino W.J., Neto R.C., Kubota L.T. (2022). Electrochemical point-of-care devices for monitoring waterborne pathogens: Protozoa, bacteria, and viruses—An overview. Case Stud. Chem. Environ. Eng..

[3] Law J.W.-F., Mutalib N.-S.A., Chan K.-G., Lee L.-H. (2015). Rapid methods for the detection of foodborne bacterial pathogens: Principles, applications, advantages and limitations. Front. Microbiol..

[4] Sagan V., Peterson K.T., Maimaitijiang M., Sidike P., Sloan J., Greeling B.A., Maalouf S., Adams C. (2020). Monitoring inland water quality using remote sensing: Potential and limitations of spectral indices, bio-optical simulations, machine learning, and cloud computing. Earth Sci. Rev..

[5] Ahmed A., Rushworth J., Hirst N.A., Millner P.A. (2014). Biosensors for whole-cell bacterial detection. Clin. Microbiol. Rev..

[6] Li J., Shen J., Qi R. (2022). Electrochemiluminescence sensing platform for microorganism detection. Biosaf. Health.

[7] Shen J., Zhou T., Huang R. (2019). Recent Advances in Electrochemiluminescence Sensors for Pathogenic Bacteria Detection. Micromachines.

[8] Zhang P., Chen Y.-P., Wang W., Shen Y., Guo J.-S. (2016). Surface plasmon resonance for water pollutant detection and water process analysis. TrAC Trends Anal. Chem..

[9] Elcin E., Öktem H.A. (2019). Whole-cell fluorescent bacterial bioreporter for arsenic detection in water. Int. J. Environ. Sci. Technol..

[10] Fricke C., Harms H., Maskow T. (2019). Rapid calorimetric detection of bacterial contamination: Influence of the cultivation technique. Front. Microbiol..

[11] Bragazzi N.L., Amicizia D., Panatto D., Tramalloni D., Valle I., Gasparini R. (2015). Chapter Six—Quartz-Crystal Microbalance (QCM) for Public Health: An Overview of Its Applications. Adv. Protein Chem. Struct. Biol..

[12] da Silva E.T.S.G., Souto D.E.P., Barragan J.T.C., de Giarola J.F., de Moraes A.C.M., Kubota L.T. (2017). Electrochemical biosensors in point-of-care devices: Recent advances and future trends. ChemElectroChem.

[13] Razmi N., Hasanzadeh M., Willander M., Nur O. (2020). Recent progress on the electrochemical biosensing of *Escherichia coli* O157: H7: Material and methods overview. Biosensors.

[14] Amiri M., Bezaatpour A., Jafari H., Boukherroub R., Szunerits S. (2018). Electrochemical methodologies for the detection of pathogens. ACS Sens..

[15] Khoshroo A., Mavaei M., Rostami M., Valinezhad-Saghezi B., Fattahi A. (2022). Recent advances in electrochemical strategies for bacteria detection. BioImpacts BI.

[16] Refaat D., Aggour M.G., Farghali A.A., Mahajan R., Wiklander J.G., Nicholls I.A., Piletsky S.A. (2019). Strategies for molecular imprinting and the evolution of MIP nanoparticles as plastic antibodies—Synthesis and applications. Int. J. Mol. Sci..

[17] Xu J., Merlier F., Avalle B., Vieillard V., Debré P., Haupt K., Tse Sum Bui B. (2019). Molecularly imprinted polymer nanoparticles as potential synthetic antibodies for immunoprotection against HIV. ACS Appl. Mater. Interfaces.

[18] Vasapollo G., Sole R.D., Mergola L., Lazzoi M.R., Scardino A., Scorrano S., Mele G. (2011). Molecularly imprinted polymers: Present and future prospective. Int. J. Mol. Sci..

[19] Bedwell T.S., Whitcombe M.J. (2016). Analytical applications of MIPs in diagnostic assays: Future perspectives. Anal. Bioanal. Chem..

[20] Piletsky S., Canfarotta F., Poma A., Bossi A.M., Piletsky S. (2020). Molecularly Imprinted Polymers for Cell Recognition. Trends Biotechnol..

[21] Pan J., Chen W., Ma Y., Pan G. (2018). Molecularly imprinted polymers as receptor mimics for selective cell recognition. Chem. Soc. Rev..

[22] Idil N., Mattiasson B. (2017). Imprinting of Microorganisms for Biosensor Applications. Sensors.

[23] Dar K.K., Shao S., Tan T., Lv Y. (2020). Molecularly imprinted polymers for the selective recognition of microorganisms. Biotechnol. Adv..

[24] Hayden O., Bindeus R., Haderspöck C., Mann K.-J., Wirl B., Dickert F.L. (2003). Mass-sensitive detection of cells, viruses and enzymes with artificial receptors. Sens. Actuators B Chem..

[25] Wangchareansak T., Thitithanyanont A., Chuakheaw D., Gleeson M.P., Lieberzeit P.A., Sangma C. (2013). Influenza A virus molecularly imprinted polymers and their application in virus sub-type classification. J. Mater. Chem. B.

[26] Wangchareansak T., Thitithanyanont A., Chuakheaw D., Gleeson M.P., Lieberzeit P.A., Sangma C. (2014). A novel approach to identify molecular binding to the influenza virus H5N1: Screening using molecularly imprinted polymers (MIPs). MedChemComm.

[27] Sangma C., Lieberzeit P.A., Sukjee W. (2017). H5N1 virus plastic antibody based on molecularly imprinted polymers. Synthetic Antibodies.

[28] Sukjee W., Thitithanyanont A., Wiboon-Ut S., Lieberzeit P.A., Paul Gleeson M., Navakul K., Sangma C. (2017). An influenza A virus agglutination test using antibody-like polymers. J. Biomater. Sci. Polym. Ed..

[29] Klangprapan S., Choke-Arpornchai B., Lieberzeit P.A., Choowongkomon K. (2020). Sensing the classical swine fever virus with molecularly imprinted polymer on quartz crystal microbalance. Heliyon.

[30] Doostmohammadi A., Youssef K., Akhtarian S., Tabesh E., Kraft G., Brar S.K., Rezai P. (2022). Molecularly imprinted polymer (MIP) based core-shell microspheres for bacteria isolation. Polymer.

[31] Akhtarian S., Doostmohammadi A., Youssef K., Kraft G., Brar S.K., Rezai P. (2023). Metal Microwires Functionalized with Cell-Imprinted Polymer for Capturing Bacteria in Water. ACS Appl. Polym. Mater..

[32] Ahmad O.S., Bedwell T.S., Esen C., Garcia-Cruz A., Piletsky S.A. (2019). Molecularly imprinted polymers in electrochemical and optical sensors. Trends Biotechnol..

[33] Sergeyeva T.A., Piletsky S.A., Brovko A.A., Slinchenko E.A., Sergeeva L.M., El’Skaya A. (1999). Selective recognition of atrazine by molecularly imprinted polymer membranes. Development of conductometric sensor for herbicides detection. Anal. Chim. Acta.

[34] Noh H., Lee J., Lee C.J., Jung J., Kang J., Choi M., Baek M.C., Shim J.H., Park H. (2019). Precise evaluation of liquid conductivity using a multi-channel microfluidic chip and direct-current resistance measurements. Sens. Actuators B Chem..

[35] Park J.K., Ryu J.C., Kim W.K., Kang K.H. (2009). Effect of electric field on electrical conductivity of dielectric liquids mixed with polar additives: DC conductivity. J. Phys. Chem. B.

[36] Heydari M.J.F., Tabatabaei N., Rezai P. (2022). Low-Cost Resistive Microfluidic Salinity Sensor for High-Precision Detection of Drinking Water Salt Levels. ACS Omega.

[37] Chon K., Moon J., Kim S., Kim S.-D., Cho J. (2007). Bio-particle separation using microfluidic porous plug for environmental monitoring. Desalination.

[38] Mei X., Yang J., Yu X., Peng Z., Zhang G., Li Y. (2023). Wearable molecularly imprinted electrochemical sensor with integrated nanofiber-based microfluidic chip for in situ monitoring of cortisol in sweat. Sens. Actuators B Chem..

[39] Karasu T., Özgür E., Uzun L. (2023). MIP-on-a-chip: Artificial receptors on microfluidic platforms for biomedical applications. J. Pharm. Biomed. Anal..

[40] Erkmen O., Erkmen M. (2021). Practice 4—Pure culture techniques. Laboratory Practices in Microbiology.

[41] Ogodo A.C., Agwaranze D.I., Daji M., Aso R.E., Egbuna C., Patrick-Iwuanyanwu K.C., Shah M.A., Ifemeje J.C., Rasul A. (2022). Chapter 13—Microbial techniques and methods: Basic techniques and microscopy. Analytical Techniques in Biosciences from Basics to Applications.

[42] DeVilbiss S.E., Steele M.K., Krometis L.-A.H., Badgley B.D. (2021). Freshwater salinization increases survival of *Escherichia coli* and risk of bacterial impairment. Water Res..

[43] Meride Y., Ayenew B. (2016). Drinking water quality assessment and its effects on residents health in Wondo genet campus, Ethiopia. Environ. Syst. Res..

[44] Mirzajani R., Kardani F. (2016). Fabrication of ciprofloxacin molecular imprinted polymer coating on a stainless steel wire as a selective solid-phase microextraction fiber for sensitive determination of fluoroquinolones in biological fluids and tablet formulation using HPLC-UV detection. J. Pharm. Biomed. Anal..

[45] Shahhoseini F., Azizi A., Bottaro C.S. (2022). A critical evaluation of molecularly imprinted polymer (MIP) coatings in solid phase microextraction devices. TrAC Trends Anal. Chem..

[46] McKnight P.E., Najab J. (2010). Mann-Whitney U Test. The Corsini Encyclopedia of Psychology.

[47] Ostovan A., Arabi M., Wang Y., Li J., Li B., Wang X., Chen L. (2022). Greenificated molecularly imprinted materials for advanced applications. Adv. Mater..

[48] Charles K.A., Matthew N.O. (2017). Fundamentals of Electric Circuits.

[49] Xu L., Ivanov P.C., Hu K., Chen Z., Carbone A., Stanley H.E. (2005). Quantifying signals with power-law correlations: A comparative study of detrended fluctuation analysis and detrended moving average techniques. Phys. Rev. E.

[50] Tokonami S., Nakadoi Y., Takahashi M., Ikemizu M., Kadoma T., Saimatsu K., Dung L.Q., Shiigi H., Nagaoka T. (2013). Label-free and selective bacteria detection using a film with transferred bacterial configuration. Anal. Chem..

[51] El Ichi S., Leon F., Vossier L., Marchandin H., Errachid A., Coste J., Jaffrezic-Renault N., Fournier-Wirth C. (2014). Microconductometric immunosensor for label-free and sensitive detection of Gram-negative bacteria. Biosens. Bioelectron..

[52] Zhang X., Wang X., Yang Q., Jiang X., Li Y., Zhao J., Qu K. (2020). Conductometric sensor for viable *Escherichia coli* and *Staphylococcus aureus* based on magnetic analyte separation via aptamer. Microchim. Acta.

[53] Qi P., Wan Y., Zhang D. (2013). Impedimetric biosensor based on cell-mediated bioimprinted films for bacterial detection. Biosens. Bioelectron..

[54] Chen Y., Wang Z., Liu Y., Wang X., Li Y., Ma P., Gu B., Li H. (2018). Recent advances in rapid pathogen detection method based on biosensors. Eur. J. Clin. Microbiol. Infect. Dis..

[55] Yilmaz E., Majidi D., Ozgur E., Denizli A. (2015). Whole cell imprinting based *Escherichia coli* sensors: A study for SPR and QCM. Sens. Actuators B Chem..

[56] Prest E.I., Hammes F., Van Loosdrecht M.C.M., Vrouwenvelder J.S. (2016). Biological stability of drinking water: Controlling factors, methods, and challenges. Front. Microbiol..

[57] Canale-Parola E. (2015). Sarcina. Bergey’s Man. Syst. Archaea Bact..

