# Supplementary material for: Microfluidic Sensor Based on Cell-Imprinted Polymer-Coated Microwires for Conductometric Detection of Bacteria in Water
Source: Biosensors (Basel). 2023 Oct 20;13(10):943. doi: 10.3390/bios13100943 (PMC10605092; doi:10.3390/bios13100943)
Supplement: Supplementary file 1 [file biosensors-13-00943-s001.zip › biosensors-2652067-supplementary.pdf]

S1. Effect of Incubation Time on Bacteria Capturing of CIP-MWs inside the Microfluidic Sensor

The fluorescent images of CIP-MWs in the microfluidic channel after running a GFP-tagged E. *coli* bacteria suspension at 10^8 CFU/mL in the time intervals of 10, 20, 30, and 40 mins are shown in Figure S1A along with the control experiment performed by running only buffer solution at the same time intervals (Figure S1B). It can be seen that, by increasing the incubation time to 30 mins, there was a significant increase in the fluorescent signals on the MWs' surface while running the bacteria suspension through the sensor. However, the experiments revealed that increasing the incubation time to 40 mins did not significantly increase the fluorescent signal. Images from the control experiment (Figure S1B) taken while running the pure buffer confirmed that the increase in the fluorescent signal while running the bacteria suspension was only due to capturing of GFP bacteria to the CIP-MW.

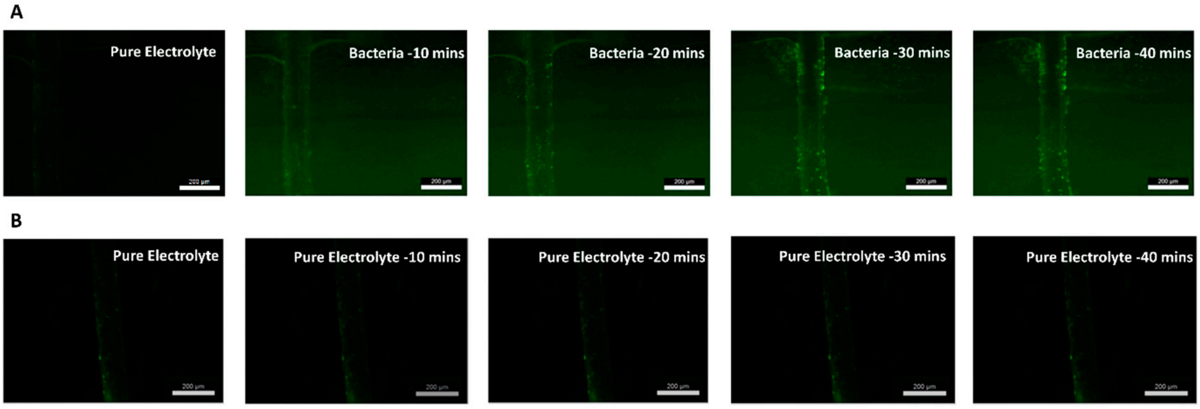

Figure S1. (A) Fluorescent images of CIP-MWs in the microfluidic channel after running buffer (first column) and bacteria suspension in 10 mins intervals. (B) Fluorescent images of CIP-MWs by running only buffer solution at the same time intervals (control experiment).

S2. Determination of Stable and Plateaued Response

The constant of proportionality between the applied current and voltage obtained by the device was resistance. This was dependent on the applied current, step duration for stabilization, type of base solution, wire material, wire coating polymer, and the geometry of the device. These parameters were kept constant among repeating trials. The device was characterized for the specific base solution (3ppm saline solution) and geometry identified in the main paper. The response curve (current vs. voltage) can be separated into two different sections. The first part (Figure S2A) had an exponential increase that was obtained as a result of the instability and very high resistance created at low current application (10 nA). The second component, highlighted in purple, comprised of the remaining curve, was linear. This linearity translated to a constant resistance value that was expected with a linear increase in current application and was the characterizing metric of the device. Plotting the resistance vs. time curve (Figure S2B), one can identify the exact moment of resistance stabilization that would result in a clear plateau, expected for the design of this device [1].

A combination of conventional and verified methods was used in the declaration of this plateau. Specifically, the 6-sigma statistics [1] and the moving average method [2] were combined to arrive at the optimized code for this application. The code can be found in Figure S3 below. This code determines the presence of a plateau in the dataset using a forward moving average function. Using a user-defined threshold, in this case <1% of the neighboring averages, we were able to determine the initial points of the plateau for our dataset.

Lastly, we calculated the representative value of the plateau. It was observed that the last 46 data points for all measurements satisfied the plateau criteria. Therefore, the value of plateau was calculated by averaging the values of the last 46 data points to obtain a consistent measurement.

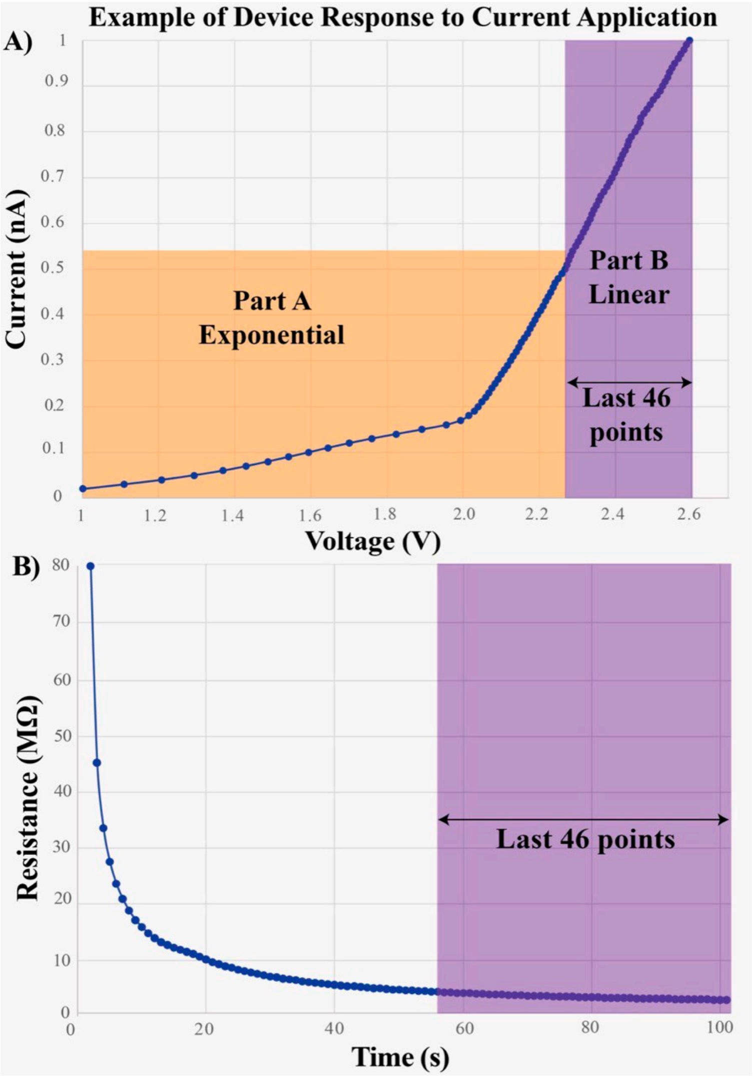

Figure S2. (A) The current vs. voltage response obtained on the Kickstart software upon application of current. (B) The corresponding resistance response of the device vs. time. Highlighted in purple is the plateau region used to determine the resistance of the device.

```matlab
excelFileName = 'Copy of April 21-MIP CONC-PLA.xlsx';
numSheets = 7;%number of sheets
numColumns = 10; % number of columns per page
% Import data from the Excel file
for j=1:numSheets
sheetData = xlsread(excelFileName, j);

%Plot the data set
for i = 1:10
    hold;
plot(sheetData(:, i+1),sheetData(:, 1))
hold;
i=i+1;
end
release;
%Calculate the moving average
% Window size for the moving average
windowSize = 5;

% Initialize an array to store the moving average
movingAverages = zeros(1, 100 - windowSize + 1);
for h=2:numColumns
transposedData = transpose(sheetData(:, h));
% Calculate the moving average
for i = 1:(101 - windowSize + 1)
    window = transposedData(i:i + windowSize - 1);
    movingAverages(i) = mean(window);
end

%Determine if the difference between the consecutive moving average is above of below
% the threshold of 1%.
for i=1:95
    if abs(movingAverages(i)-movingAverages(i+1))/(movingAverages(i))<0.01
      if i+floor(windowSize/2)<30
         i=i+1;
      else
          if i+floor(windowSize+1/2)>56
              fprintf("plateau not reached");
          end
           i+floor(windowSize/2)
        break
       end

    else
        i=i+1;
    end
end
end
```

Figure S3. MATLAB code used to determine the initial plateau.

References

[1] M. J. Farshchi Heydari, N. Tabatabaei, and P. Rezai, "Low-Cost Resistive Microfluidic Salinity Sensor for High-Precision Detection of Drinking Water Salt Levels.," *ACS omega*, vol. 7, no. 18, pp. 15529–15539, May 2022, doi: 10.1021/acsomega.2c00268.

[2] L. Xu, P. C. Ivanov, K. Hu, Z. Chen, A. Carbone, and H. E. Stanley, "Quantifying signals with power-law correlations: A comparative study of detrended fluctuation analysis and detrended moving average techniques," *Phys. Rev. E*, vol. 71, no. 5, p. 51101, May 2005, doi: 10.1103/PhysRevE.71.051101.
